# Regulatory role of TIPE2 by inhibiting microglial pyroptosis and glycolysis in the treatment of sepsis‐associated encephalopathy

**DOI:** 10.1002/ctm2.70881

**Published:** 2026-11-06

**Authors:** Min Yuan, Yunzhao Yang, Huifan Liu, Qian Kong, Guoqing Jing, Hailong Gong, Yuxuan Wang, Zhengyu Zhang, Wanhong Liu, Xuemin Song, Zhongyuan Xia, Xiaojing Wu

**Affiliations:** ^1^ Department of Anesthesiology Renmin Hospital of Wuhan University Wuhan Hubei China; ^2^ Research Centre of Anesthesiology and Critical Care Medicine Zhongnan Hospital of Wuhan University Wuhan Hubei China; ^3^ School of Pharmaceutical Sciences Wuhan University Wuhan Hubei China; ^4^ Department of Immunology School of Basic Medical Sciences Wuhan University Wuhan Hubei China; ^5^ Department of Anesthesiology Hainan General Hospital (Hainan Affiliated Hospital of Hainan Medical University) Haikou China

**Keywords:** glycolysis, microglia, pyroptosis, sepsis‑associated encephalopathy, TIPE2

## Abstract

**Background:**

Sepsis_associated encephalopathy (SAE) denotes acute cerebral dysfunction triggered by sepsis and is accompanied by high mortality. Our previous study showed that TIPE2 significantly ameliorated cognitive impairment in SAE mice by inhibiting neuroinflammation, but the specific mechanism was unknown.

**Methods:**

TIPE2 expression and cell specificity were analysed by scRNA‑seq and immunofluorescence. To establish SAE, cecal ligation and puncture (CLP) was carried out in TIPE2 knockout (KO) and WT mice. Neuronal/synaptic damage was assessed by TUNEL and TEM; cognitive function by behavioural tests and EEG. Hippocampal scRNA‑seq compared WT and TIPE2 KO mice. In vitro, primary microglia and BV2 cells were stimulated with LPS/nigericin. Protein expression (Western blot/IF), RhoA activation (GST‑pulldown), glucose/lactate levels, ROS production, and TIPE2‑RhoA interaction (Co‑IP/confocal) were examined.

**Results:**

scRNA‑seq confirmed that TIPE2 is predominantly expressed in microglia and downregulated in SAE; its deficiency promotes pro‑inflammatory microglia associated with pyroptosis and glycolysis. TIPE2 KO significantly aggravated cognitive impairment, promoted M1 polarisation, enhanced pyroptosis (GSDMD cleavage, caspase‑1 activation), and increased ROS production. Mechanistically, TIPE2 directly bound to RhoA and inhibited its activation, thereby suppressing NLRP3‑mediated pyroptosis and glycolysis in microglia. Treatment with the RhoA‑specific inhibitor Rhosin reversed TIPE2 deficiency‑induced microglial polarisation, pyroptosis, ROS production, and glycolytic reprogramming, and also ameliorated cognitive dysfunction in TIPE2 KO mice.

**Conclusions:**

TIPE2 protects against SAE by directly interacting with RhoA, inhibiting its activation, and subsequently suppressing NLRP3‑mediated microglial pyroptosis and glycolysis.

## Key points

- TIPE2 deficiency exacerbates synaptic damage and cognitive dysfunction in SAE mice.
- TIPE2 directly binds to RhoA and inhibits its activation, thereby suppressing NLRP3‑mediated microglial pyroptosis and glycolytic reprogramming.
- Pharmacological inhibition of RhoA activation significantly reversed both enhanced microglial pyroptosis and glycolysis induced by TIPE2 deficiency.

## Graphical abstract

TIPE2 inhibits the activation of RhoA by interacting with RhoA, subsequently regulating NLRP3‐mediated pyroptosis and glycolysis in microglia and play a protective role in SAE.

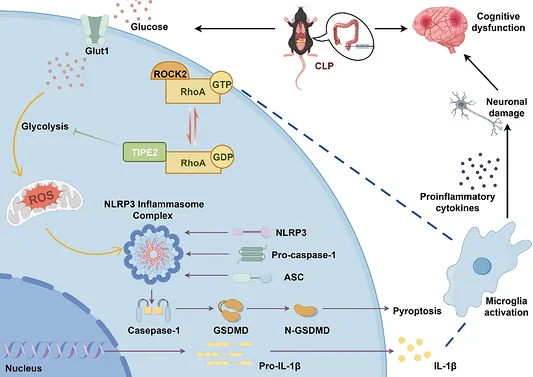

## 1 INTRODUCTION

Sepsis refers to a potentially fatal condition characterised by organ dysfunction, resulting from a dysregulated host response to infection‐induced injury, which accounting for 54.4% of global deaths annually.^1^ Up to 50% of sepsis causes acute progressive brain dysfunction sepsis‐related encephalopathy (SAE), which is characterised by diffuse brain dysfunction with significant cognitive and memory impairment.^2^ The worsening neuroinflammation and brain injury development are crucial factors in the pathology of severe sepsis, impacting survival and prognosis significantly.^3^ Despite substantial progress in fundamental theoretical research and exploration of molecular mechanisms underlying sepsis, its pathological progression remains difficult to curb clinically. To date, the pathophysiological mechanisms and strategies for the prevention and treatment of SAE have not been clarified, remaining a vital scientific problem that demands an urgent solution in both basic and clinical disciplines.

Within the central nervous system (CNS), microglia serve as the earliest responders to pathogenic insults and are indispensable for preserving brain homeostasis.^4^ The initiation of the inflammatory response is closely related to the transcription and assembly of inflammasomes, with NLRP3 being the most abundant inflammasome in CNS and a key point for microglial cells activation and neuroinflammatory responses. According to previous studies, pyroptosis driven by the NLRP3/Caspase‐1/GSDMD signalling cascade serves as a critical component of neuroinflammation induced by activated microglia.^5^ Therefore, inhibiting microglial cells pyroptosis may be a good strategy to prevent excessive neuroinflammatory responses during sepsis. Glucose is an essential fuel for microglial cells, and under inflammatory conditions, microglial cells promote glucose uptake and increase cellular glycolysis.^6^ Recent studies indicate that Glut1 amplifies NLRP3 inflammasome activation and is involved in controlling IL‐1β release.^7^ Additionally, Renaudin et al. found that televated Glut1 expression results in higher IL‐1β levels.^8^ Therefore, blocking glycolysis is one of the important factors for controlling microglial pyroptosis.

TIPE2, which belongs to the TNFAIP8 family, negatively modulates innate and adaptive immune responses and plays a critical role in immune tolerance.^9^ Our previous research also found that overexpression of TIPE2 effectively inhibits lung tissue inflammation in septic mice.^10^ Additionally, TIPE2 suppresses dendritic cell autophagy and further negatively regulates dendritic cell immune function.^11^ Current research on TIPE2 in CNS injuries mainly focuses on stroke, where it has been found to exert protective effects by inhibiting inflammation and preventing brain tissue damage due to excessive inflammatory responses, the release of TIPE2 can suppress brain tissue damage caused by excessive inflammatory responses during acute cerebral ischemia.^12^ Our preliminary findings suggest that TIPE2 can real reduce neuroinflammation in SAE by inhibiting NF‐κB signalling in microglia, which indicating the involvement of TIPE2 in SAE neuroinflammation.^13^ Our previous work established a correlative link between TIPE2 and neuroinflammation, but the specific mode of action and its broader functional implications remained unclear. This study sought to clarify the regulatory function and underlying mechanism of TIPE2 in SAE, and to explore its role in the novel contexts of pyroptosis and glycolysis using multiple molecular biological methods, thereby proposing new potential strategies for the early intervention and management of SAE.

## 2 METHOD

### 2.1 Animal models of SAE

Male C57BL/6 mice in adulthood (6–8 weeks old, weighing 20–25 g) were acquired from the Animal Experiment Center of Hubei Province and TIPE2 gene knockout mice (TIPE2 KO) were generated by Wuhan Xianran Biological Technology Co. LTD. These animals were accommodated in the animal facility of Wuhan University. There, they were kept under controlled conditions with stable temperature and humidity, and received food and water without restriction. The mouse SAE model was induced through CLP, following the method reported earlier.^14^ Anaesthesia was achieved by intraperitoneal injection of pentobarbital sodium at 50 mg/kg. After shaving and disinfecting the abdomen, a 1–2 cm cut was created in the right quadrant. The cecum was exposed, tied off using 4‐0 silk suture, and then pierced twice using a 21‐G needle so that a small amount of fecal material would leak. Subsequently, the cecum was placed back, and the wound was sutured closed. Every mouse was given warm saline subcutaneously (50 mL/kg) to support fluid resuscitation and were kept warm using a heating blanket. After regaining consciousness, the animals were put back into their cages for postoperative care.

### 2.2 Electroencephalography (EEG)

EEG was used to record brain activity in unrestrained mice. Once anaesthetised, the skull was revealed through a midline incision in the scalp. Subsequently, burr holes were made, and screw electrodes were placed and then fixed using dental cement. One week after implantation, CLP surgery was performed, and EEG signals were recorded 24 h later using a bioelectrical amplifier. Data were processed and analysed with Labchart software.

### 2.3 Morris water maze test (MWM)

A circular tank was used for the water maze, containing a submerged platform positioned in the southwest quadrant. The water surface was kept 1 cm higher than the platform, with the temperature held at 23°C. During the initial four days, every animal had 60 s to find the platform, and escape latency was documented. On day 5, the platform the platform was taken out, then the mice were placed in the opposite quadrant for a 60 s free swim. The amount of time spent within the target quadrant and how often mice crossed the platform were recorded.

### 2.4 Transmission electron microscopy (TEM)

Hippocampal ultrathin slices were mounted onto copper grids (200 mesh), double‐stained using 4% uranyl acetate plus 0.04% lead citrate, and then examined under TEM. The postsynaptic density (PSD) thickness was determined by measuring the perpendicular span between the postsynaptic membrane and the convex apex of the synaptic complex. The width of the synaptic cleft was calculated as the mean of its narrowest and widest measurements.

### 2.5 TUNEL staining

TUNEL staining was performed according to the kit instructions. Brain sections slices were visualised using confocal microscope. To evaluate apoptosis, the proportion of TUNEL‐positive cells was calculated across three randomly chosen fields per section.

### 2.6 Cell culture and treatment

We acquired BV2 microglial cell line from the Cell Bank of the Chinese Academy of Sciences (No. SCSP‐5208) and primary microglia were obtained from 1‐day‐old wild‐type and TIPE2 KO pups. Cells were exposed to 1 µg/mL LPS (E. coli 0111: B4, L2630, Sigma) or pre‐incubated with 20 µM Rhosin (HY‐12646, MedChemExpress) before LPS stimulation. Plasmids and siRNAs were transfected using Lipofectamine 3000 (Invitrogen, L3000‐015). The plasmid Myc‐TIPE2 and HA‐RhoA were generated by Wuhan MiaoLing Plasmid Platform. siTIPE2 and siNC were procured from Tsingke Biotechnology (Nanning, China). After a 48‐h period, both the cells and supernatants were harvested. The primer sequences are provided in Table 1.

**TABLE 1:** Primers for siTIPE2.

Primer name	Primer sequences
siTIPE2 forward primer	GCAGCGAGGUGCUAGACGATT
siTIPE2 reverse primer	UCGUCUAGCACCUCGCUGCTT

### 2.7 Enzyme‐linked immunosorbent assay (ELISA)

Serum TNF‐α (RK00027, ABclonal), IL‐1β (RK00006, ABclonal), IL‐18 (RK00104, ABclonal), and IL‐6 (RK00008, ABclonal) were assayed with ELISA kits obtained from R&D Systems (USA), according to the protocol provided by the manufacturer.

### 2.8 Reactive oxygen species assay (ROS)

Intracellular levels of ROS were determined with a DCFH‐DA probe (S0033S, Beyotime). Following treatment, the cells underwent washing and incubation with DCFH‐DA in DMEM, following the manufacturer's protocol, and were subsequently subjected to flow cytometry.

### 2.9 Real‐time quantitative PCR (RT‐qPCR)

RNA was isolated with TRIzol reagent (15596‐026, Invitrogen) and subsequently converted into cDNA through reverse transcription. Gene expression levels were examined by RT‐qPCR. Relative quantification was carried out with the 2^–ΔΔCt^ approach, using GAPDH as the internal reference. Table 2 presents the primer sequences.

**TABLE 2:** Primers for qRT‐PCR analyses of the relevant sequences.

Primer name	Primer sequences
Mouse‐TNF‐α forward primer	GGCATGGATCTCAAAGACAAC
Mouse‐TNF‐α reverse primer	TGGATGCTCTCATCAGGACAG
Mouse‐IL‐1β forward primer	GAAATGCCACCTTTTGACAGTG
Mouse‐IL‐1β reverse primer	TGGATGCTCTCATCAGGACAG
Mouse‐IL‐6 forward primer	CATGTTCTCTGGGAAAT.CGTGG
Mouse‐IL‐6 reverse primer	GTACTCCAGGTAGCTATGGTAC
Mouse‐GAPDH forward primer	AGGTCGGTGTGAACGGATTTG
Mouse‐GAPDH reverse primer	TGTAGACCATGTAGTTGAGGTA

### 2.10 Western blot analysis

Hippocampal tissue samples and cultured cells underwent lysis using RIPA buffer supplemented with PMSF and a phosphatase inhibitor (ST506, Beyotime). Protein levels were quantified with a BCA assay kit (P0011, Beyotime). Subsequently, equal quantities of denatured proteins were resolved via SDS–PAGE, electroblotted onto PVDF membranes, and incubated with 5% BSA for 2 h at room temperature to block nonspecific binding. Finally, the membranes were probed with primary antibodies: TIPE2 (E5Q2S, CST), RhoA (ab187027, Abcam), NLRP3 (15101, CST), GSDMD (39754, CST), Caspase‐1 (AG‐20B‐0042, Adipogen), HA (51064‐2‐AP, Proteintech), Myc (60003‐2‐Ig, Proteintech), PSD95 (3409, CST), SYP (AF0257, Affinity), Glut1 (A6982, Abclonal), and HK2 (22029‐1‐AP, Proteintech). Following TBST rinses, the blots were probed with appropriate secondary antibodies conjugated to HRP. Target bands were detected via enhanced chemiluminescence, and their intensities were quantified through densitometric analysis using ImageJ. Data were expressed as relative intensity versus controls.

### 2.11 RhoA activation assay

The activation of RhoA (GTP‐RhoA) was assessed using a pull‐down assay kit (BK036, Cytoskeleton, USA). In brief, cell lysates collected on the ice were mixed with GST‐Rhotekin‐RBD beads by rotating them in a 4°C refrigerator to capture GTP‐RhoA. Following washes, the beads were resuspended with Laemmli buffer, heated, and then subjected to SDS‐PAGE and Western blot analysis.

### 2.12 Co‐immunoprecipitation

Cell lysis was carried out using NP‐40 buffer (HY‐K1002, MCE) on ice for 30 min. After centrifugation, the supernatant fractions were mixed with the primary antibody and rotated overnight at 4°C with rotation. Subsequently, magnetic beads (HY‐K0202, MCE) conjugated with Protein A/G were introduced and incubated for 4 h. After three washes, the beads were re‐suspended in SDS‐PAGE loading buffer, boiled for 10 min at 100°C, and then subjected to Western blot analysis.

### 2.13 Immunofluorescent staining

Cell samples were first fixed in 4% paraformaldehyde, then permeabilised using 0.3% Triton X‐100, and blocked with 5% BSA for 60 min at 37°C. Next, the cells were probed with the following primary antibodies: Arg1 (16001‐1‐AP, Proteintech), iNOS (22226‐1‐AP, Proteintech), Iba‐1 (ab178846, Abcam), TIPE2 (E5Q2S, CST), ASC (AG‐25B‐0006, Adipogen), RhoA (ab187027, Abcam), and GTP‐RhoA (26904, New East Bioscience). After rinsing, secondary antibodies (Abbkine, USA) were applied, followed by DAPI counterstaining and confocal microscopy imaging.

### 2.14 ScRNA‐seq library construction and sequencing

Hippocampal cells were harvested, dissociated, and counted. Subsequently, these cells were combined with reverse transcription reagents, partitioning oil, and barcoded hydrogel beads within a SeekOne® chip, thereby producing emulsion droplets. Reverse transcription then took place, which was followed by cDNA purification, amplification, and library preparation. Finally, the libraries were subjected to purification, quality assessment, and sequencing using the Illumina NovaSeq 6000 sequencing platform (PE150).

### 2.15 ScRNA‐seq data analysis

Analyses of single‐cell profiles were conducted in the R environment with Seurat (v4.4.0), which was employed for clustering and dimensionality reduction. Cells of poor quality were excluded according to mitochondrial percentage as well as UMI and gene counts. DoubletFinder (v2.0.4) was applied to eliminate doublets, while Canonical Correlation Analysis (CCA) served to correct batch effects. FindAllMarkers was used to discover marker genes, and cell types were assigned via SingleR together with CellMarker 2.0. Seurat's FindMarkers facilitated differential expression analysis, with results displayed using ggplot2, ggpubr, and scRNAtoolVis. Enrichment analyses for KEGG and GO pathways were carried out with clusterProfiler. Microglial subsets were re‐clustered into SAM, HM, and NAM subclusters.

### 2.16 Measurement of glucose and lactate levels

Commercial kits obtained from Nanjingjiancheng Technology Co., Ltd. were employed to quantify glucose and lactate levels. Glucose content was determined by standard curve and normalised to protein concentration. For lactate, both standards and sample supernatants were pipetted into 96‐well plates, kept at 37°C for half an hour, after which optical density was recorded at 530 nm. The concentration of lactate was derived from the standard curve.

### 2.17 Statistical analysis

Data are presented as mean ± SEM. For the training phase of the MWM test, a repeated‐measures two‐way ANOVA was employed, and Bonferroni adjustment was applied to account for multiple comparisons. For comparisons among multiple groups, a two‐way ANOVA was conducted, followed by Bonferroni post hoc testing. Statistical significance was defined as *p* < .05.

## 3 RESULT

### 3.1 TIPE2 primarily expressed in microglia and suppresses SAE inflammatory responses

To investigate the transcriptional changes induced by SAE in mouse brain, we re‐analysed scRNA‐seq data from the GSE211099 dataset (PBS control vs. LPS‐treated). Through unsupervised clustering, UMAP dimensionality reduction, and cell‑type annotation using specific marker genes, we identified major cell populations, including astrocytes, endothelial cells, microglia, neurons, oligodendrocytes, oligodendrocyte precursor cells (OPCs), and pericytes (Figure 1A). The UMAP plots showed similar distributions of cell populations between LPS‑treated and PBS control groups, indicating that LPS treatment did not grossly alter the major cellular composition (Figure 1B). The heatmap (Figure 1C) displays two main clusters of upregulated and downregulated genes after LPS treatment. Among the downregulated genes, we focused on *Tnfaip8l2* (*TIPE2*), a known negative regulator of innate and adaptive immunity critical for maintaining immune tolerance. Given the central role of microglia in neuroinflammation, we assessed TIPE2 expression specifically in this cell type. Violin plot analysis (Figure 1D) showed that TIPE2 expression in microglia was significantly decreased in the LPS‑treated group compared with the PBS control. To determine the cellular expression profile of TIPE2, we analysed its expression across all identified cell types. Dot plot analysis (Figure 1E) revealed that TIPE2 exhibited high cell‑type specificity, being almost exclusively enriched in microglia, as indicated by high average expression level (dot colour) and high percentage of expressing cells (dot size). Data split by treatment group (PBS vs. LPS) (Figure 1F) further confirmed this specificity and showed that TIPE2 downregulation occurred predominantly within the microglial population.

**FIGURE 1:**
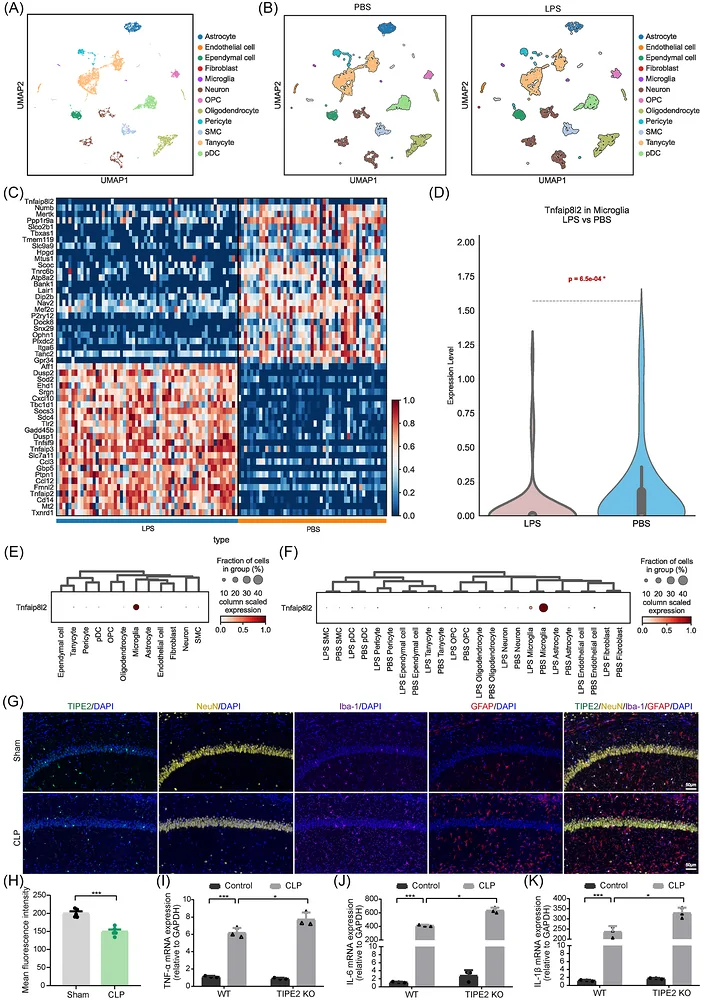
TIPE2 primarily expressed in microglia and suppresses SAE inflammatory responses. (A) Uniform Manifold Approximation and Projection (UMAP) dimensional reduction plot displaying the major cell clusters identified from the GSE211099 dataset, coloured according to cell type annotation. (B) UMAP plots showing the distribution of cell clusters from (A), split by treatment condition (left: phosphate‐buffered saline (PBS)‐treated group; right: lLipopolysaccharide (LPS)‐treated group). (C) Heatmap of differentially expressed genes (DEGs) comparing the gene expression profiles between the LPS group and the PBS group. Rows represent genes, and columns represent individual cells. (D) Violin plot illustrating the distribution of Tnfaip8l2 (TIPE2) expression levels in microglia between the PBS group and the LPS group. (E) Dot plot showing the expression profile of TIPE2 across all identified cell types. The size of the dot represents the proportion of cells within the cluster expressing the gene, and the colour intensity reflects the average expression level. (F) Dot plot displaying the expression of TIPE2, further split by both cell type and treatment condition (PBS vs. LPS). (G) Immunofluorescence analysis of TIPE2 expression and its colocalisation with various neuronal cells. (H) Mean fluorescence intensity of TIPE2 in the hippocampus of WT and CLP mice (*n* = 3 per group). (I–K) Levels of *TNF‐α*, *IL‐6* and *IL‐1β* in hippocampal tissue were measured by qRT‐PCR (*n* = 3 per group). *p < .05, ***p < .001.

We next validated these findings at the animal level using immunofluorescence staining. Colocalisation analysis of TIPE2 with NeuN (neuronal marker), Iba‑1 (microglial marker), and GFAP (astrocyte marker) showed that TIPE2 mainly colocalised with Iba‑1, confirming its predominant expression in microglia (Figure 1G). Quantification of TIPE2 fluorescence intensity in hippocampal tissue revealed a significant reduction in CLP group relative to Sham group (Figure 1H), consistent with the scRNA‑seq results and further suggesting that TIPE2 may be involved in SAE development and progression. Finally, mRNA levels of proinflammatory cytokines (*TNF‐α*, *IL‐6*, and *IL‐1β*) were measured in the mouse hippocampus. Relative to the Sham group, cytokine expression was significantly elevated in the CLP group, and this upregulation was further promoted by TIPE2 deficiency under CLP challenge (Figure 1I‐K).

### 3.2 TIPE2 deficiency aggravates CLP‐induced brain damage and promotes microglial activation in SAE mice

To examine neuro‐excitability, we monitored the brain electrical activity of mice using EEG (Figure 2A). EEG monitoring revealed that CLP modelling significantly reduced brain electrical activity, characterised by decreased α and β wave frequencies and increased δ wave frequency (Figure 2B), confirming successful SAE induction. TIPE2 knockout further exacerbated this suppression. Transmission electron microscopy of hippocampal synapses showed that CLP reduced synaptic density, postsynaptic density thickness, and synaptic vesicle number, while widening the synaptic cleft and causing mitochondrial swelling. TIPE2 deficiency aggravated these ultrastructural damages (Figure 2C and D). Western blot analysis demonstrated that CLP decreased the expression of synapse‑associated proteins PSD95 and synaptophysin (SYP), with TIPE2 knockout further reducing their levels (Figure 2E–G). In the Morris water maze, CLP mice exhibited longer escape latencies during the 4‑day training period than WT mice, and TIPE2 deficiency further prolonged escape latency (Figure 2I). In the spatial probe test on day 5, CLP mice showed reduced platform crossings and less time in the target quadrant, effects intensified by TIPE2 knockout (Figure 2J and K). Immunofluorescence analysis of microglial activation revealed that CLP enlarged microglial cell bodies and increased Iba‑1 fluorescence intensity, indicating microglial activation, which was further enhanced in TIPE2‑deficient mice (Figure 2L and M). These results suggest that TIPE2 knockout aggravates sepsis‑induced synaptic and neuronal damage, likely through enhanced neuroinflammation and microglial activation.

**FIGURE 2:**
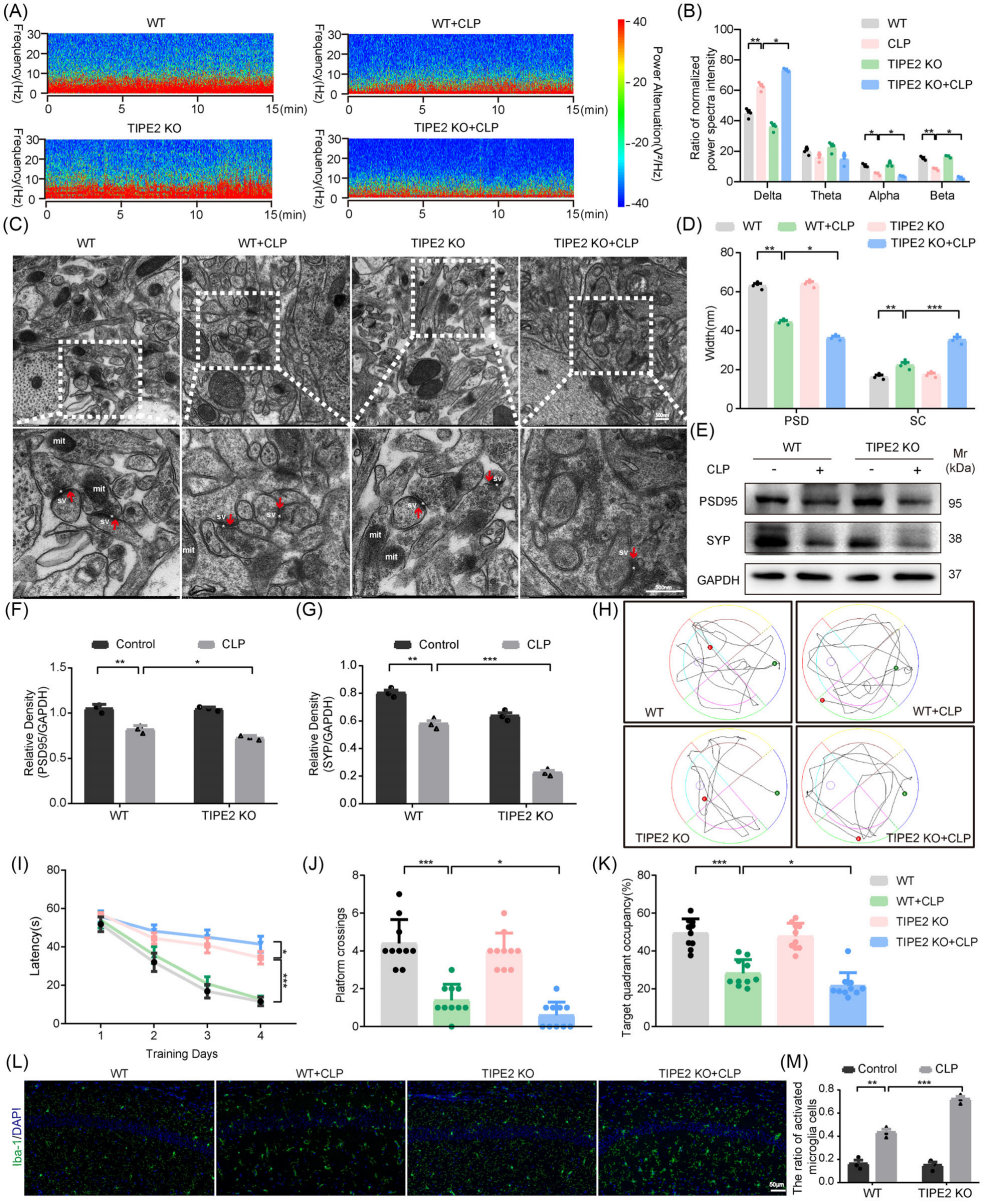
TIPE2 deficiency aggravates CLP‐induced brain damage and promotes microglial activation in SAE mice. (A) Mouse EEG power spectrum. (B) Mean power percentage of mouse EEG for δ, θ, α, and β waves (*n* = 5 per group). (C) Ultrastructural changes in hippocampal synapses of each group under transmission electron microscopy. Scale bar, 500 nm. (D) Statistical results of postsynaptic compact thickness and synaptic cleft width in hippocampus of each group (*n* = 3 per group). (E, G) Levels of PSD95 and SYP protein were measured by western blot (*n* = 3 per group). (H) Representative swim traces of each group in the space exploration experiment on the 5th day (*n* = 10 per group). (I) Target quadrant occupancy. The proportion of time spent in the target quadrant in the total time on the 5th day (*n* = 10 per group). (J) Platform crossings. Times of crossing the platform on the 5th day (*n* = 10 per group). (K) Latency. The latency of each group in MWM tests for four consecutive days (*n* = 10 per group). (L) Microglia activation was assessed by immunofluorescence. Scale bar, 50 µm. (M) The ratio of activated microglia cells (*n* = 3 per group). *p < .05, **p < .01, ***p < .001.

### 3.3 TIPE2 is closely associated with pyroptosis and glycolysis in microglia of SAE mice

To investigate the role of TIPE2 in microglial functional dynamics during SAE, we performed scRNA‐seq on hippocampal cells from WT and TIPE2 KO mice after CLP‐induced sepsis. Cell‐type‐specific signature genes validated each cluster, and the relative proportions of these cell types differed between WT+CLP and TIPE2 KO+CLP groups (Figure 3A–D). Focusing on microglia, subpopulation analysis based on distinct gene expression profiles revealed three subsets (Figure 3E): Neuro‑Regulatory Associated Microglia (NAM), Homeostatic Microglia (HM), and Sepsis‐Associated Microglia (SAM). Signature gene analysis (Figure 3F) confirmed their functional identities: NAM highly expressed neural homeostasis genes (e.g., *Ttr*, *Ptgds*); HM expressed homeostatic genes (e.g., *Hspa1a*, *Txnip*); and SAM exhibited elevated inflammatory genes (e.g., *Il1b*) and monocyte chemokines (e.g., *Cxcl2*, *Ccl3*), indicating a pro‑inflammatory phenotype. Comparison of microglial subpopulation proportions between WT+CLP and TIPE2 KO+CLP (Figure 3G) showed a marked increase in the SAM subset in TIPE2 KO mice, suggesting that TIPE2 deficiency promotes the generation or maintenance of pro‑inflammatory microglia. Transcriptomic analysis was conducted to compare WT_CLP and TIPE2KO_CLP mice. A volcano plot (Figure 3H) was generated to visualise differentially expressed genes (DEGs), with the screening thresholds set at |log_2_FC| ≥ 0.5 and *p* < 10–^2^ ^0^. Transcriptomic comparison demonstrated that the TIPE2 KO+CLP group had markedly higher expression of several transcripts, including *Nlrp3*, *Hk2*, *Rock2*, *Cxcl2*, and *Il1b*. This finding implies that losing TIPE2 could stimulate signalling cascades connected to glycolysis and pyroptosis. Indeed, when we assessed cellular pyroptosis using a pathway gene set and visualised it with violin plots (Figure 3I), the SAM subpopulation in TIPE2 KO+CLP mice showed significantly higher pyroptosis scores than in WT+CLP mice, whereas NAM and HM subsets were unchanged. This finding aligns with the upregulation of pyroptosis‑associated genes (e.g., *Nlrp3*) observed in the volcano plot (Figure 3H), collectively suggesting that TIPE2 deficiency contributes to SAE pathophysiology by promoting pyroptosis in the SAM subpopulation. Lastly, TUNEL assays performed on hippocampal sections (Figure 3J) showed obvious apoptosis in response to CLP. Moreover, apoptotic cell counts were markedly increased within TIPE2 KO+CLP animals relative to WT+CLP animals (Figure 3K).

**FIGURE 3:**
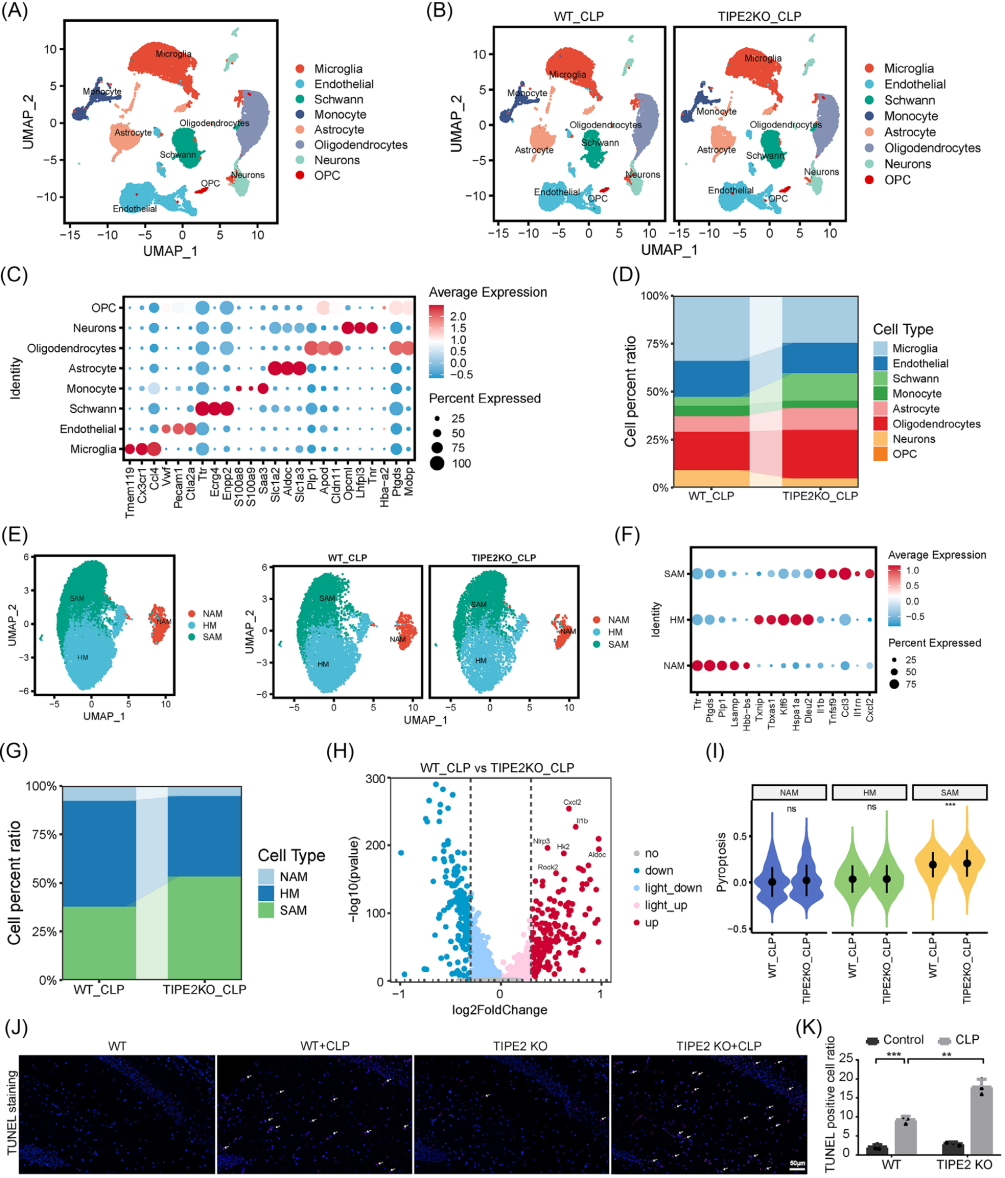
TIPE2 is closely associated with pyroptosis and glycolysis in microglia of SAE mice. (A, B) UMAP plot of unsupervised clustering identifying major cell types in hippocampal tissues from CLP‐induced septic mice. (C) Dot plot showing the average expression and percentage of cells expressing marker genes for each identified cell type, validating cell type annotations. (D) Stacked bar plot illustrating the percentage ratio of each cell type in WT+CLP and TIPE2 KO+CLP groups. (E) UMAP plots of microglial subset clustering (left) and the distribution of microglial subsets in WT+CLP and TIPE2 KO+CLP groups (right). (F) Dot plot depicting the average expression and percentage of cells expressing signature genes for three microglial subsets: neuroregulation‐associated microglia (NAM), homeostatic microglia (HM), and sepsis‐associated microglia (SAM). (G) Stacked bar plot showing the percentage ratio of microglial subsets in WT+CLP and TIPE2 KO+CLP groups. (H) Volcano plot of differentially expressed genes (DEGs) in microglia between WT+CLP and TIPE2 KO+CLP groups. (I) Violin plots showing the pyroptosis levels in different microglial subsets (NAM, HM, SAM) between WT+CLP and TIPE2 KO+CLP groups. (J) TUNEL staining. Scale bar, 50 µm. (K) Proportion of apoptosis in the hippocampus of each group in TUNEL staining (*n* = 3 per group). **p < .01, ***p < .001.

### 3.4 TIPE2 deficiency aggravates activation and pyroptosis of microglia

To determine whether the exacerbated neural damage in TIPE2 knockout mice is related to microglial pyroptosis, we isolated primary microglia from WT as well as TIPE2 KO mice. As shown in Figure 4A and B, TIPE2 expression was reduced in the WT+LPS/Nigericin group, while the levels of NLRP3, Caspase‐1 p20, and GSDMD‑N were significantly increased. Compared with the WT+LPS/Nigericin group, the TIPE2 KO+LPS/ Nigericin group showed further upregulation of these proteins associated with pyroptosis such as NLRP3, Caspase‐1 p20 and GSDMD‑N, together with greater release of IL‐1β, IL‐18, IL‐6, and TNF‐α. To further validate the role of TIPE2 in microglial pyroptosis, BV2 cells were transfected with siTIPE2 or TIPE2‑overexpressing plasmid. When exposed to LPS plus nigericin, silencing TIPE2 led to a pronounced rise in pyroptosis‐associated markers (NLRP3, Caspase‐1 p20, GSDMD‐N), promoted M1 polarisation, and secreted more pro‐inflammatory cytokines. In contrast, TIPE2 overexpression markedly attenuated these effects (Figure 4C, D, H, and I). Immunofluorescence analysis revealed that ASC oligomerisation, a hallmark of inflammasome activation, was more pronounced in the TIPE2 KO+LPS/Nigericin group (Figure 4F). Furthermore, after LPS/Nigericin treatment, the M1 marker iNOS was significantly upregulated while the M2 marker Arg1 was downregulated, and this polarisation imbalance was further exacerbated by TIPE2 knockout (Figure 4G). Consistently, flow cytometry analysis showed that TIPE2 knockdown further elevated LPS/nigericin‑induced reactive oxygen species (ROS) generation, whereas TIPE2 overexpression suppressed this increase (Figure 4J and K). Quantitative statistical analysis plots of western blot data in Figure S2.

**FIGURE 4:**
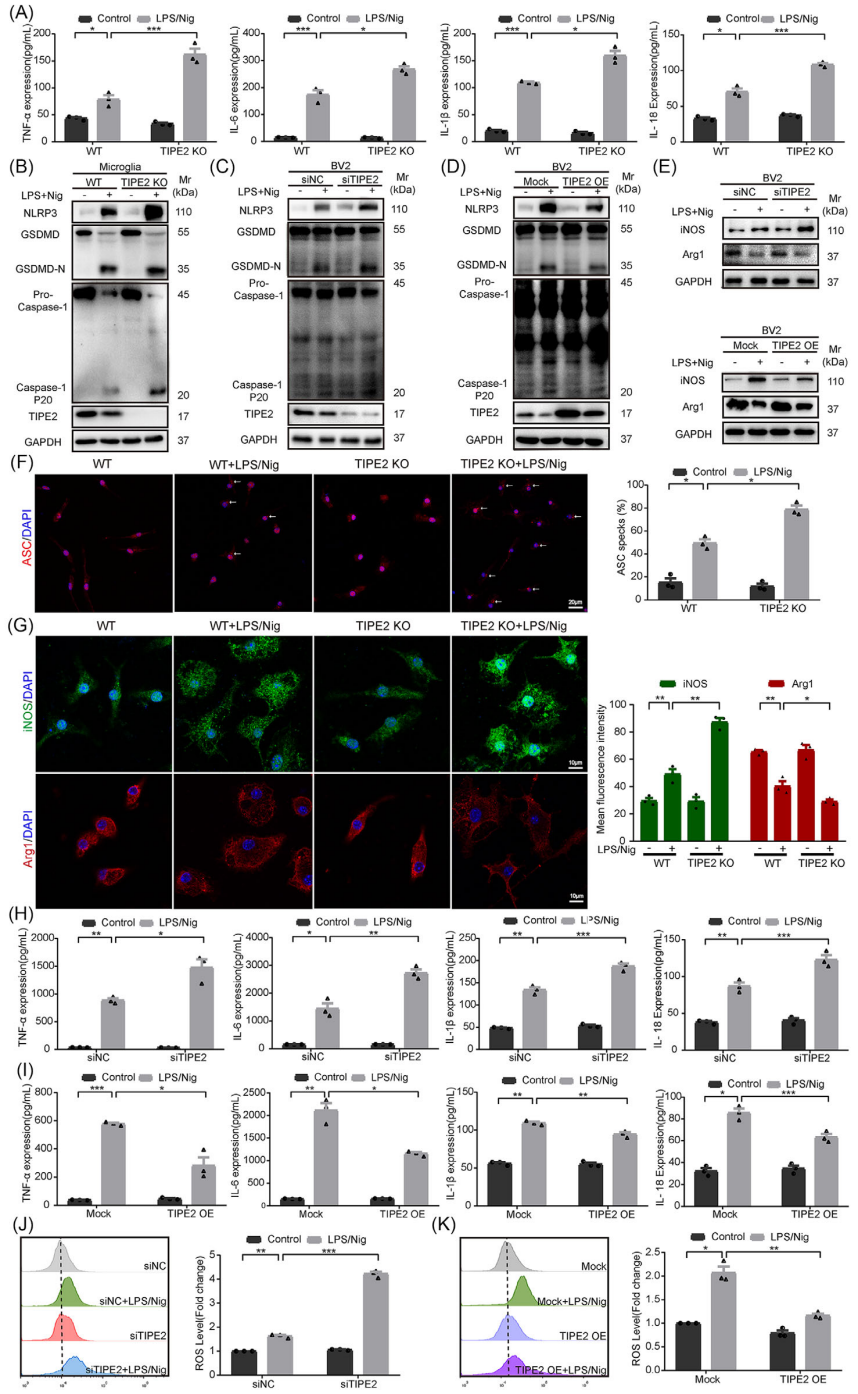
TIPE2 deficiency aggravates activation and pyroptosis of microglia. (A) Levels of TNF‐α, IL‐6, IL‐18 and IL‐1β in primary microglia supernatant were measured by ELISA. (B) The expression of TIPE2, NLRP3, Caspase‐1 P20, GSDMD‐N, iNOS and Arg1 in primary microglia was determined by western blot. (C, D) The expression of TIPE2, NLRP3, Caspase‐1 P20, GSDMD‐N were determined after transfection of siTIPE2 and pCMV‐Myc‐TIPE2 plasmid in BV2 microglia by western blot. (E) The expression of iNOS and Arg1 in BV2 microglia was determined by western blot. (F) ASC specks were visualised by immunofluorescence. Scale bar, 20 µm. (G) The expression of microglial polarisation markers (iNOS and Arg1) in primary microglia were assessed by immunofluorescence. Scale bar, 10 µm. (H, I) Levels of TNF‐α, IL‐6, IL‐18 and IL‐1β in BV2 microglia supernatant after transfection of siTIPE2 and pCMV‐Myc‐TIPE2 plasmid were measured by ELISA. (J) Levels of ROS in BV2 microglia after transfection of siTIPE2. (K) Levels of ROS in BV2 microglia after transfection of pCMV‐Myc‐TIPE2 plasmid. *n* = 3 per group, *p < .05, **p < .01, ***p < .001.

### 3.5 TIPE2 interacts with RhoA to inhibit RhoA/Glut1‐driven glycolysis

Following this, we employed immunofluorescence to map the subcellular distribution of RhoA and TIPE2. Figure 5A illustrates that TIPE2 and RhoA exhibited obvious co‑localisation. To investigate the molecular interaction between them, we co‑transfected HEK293T cells with pCMV‑Myc‑TIPE2 and pCAGGS‑HA‑RhoA and performed exogenous co‑immunoprecipitation (Co‑IP). The interaction was further validated by endogenous Co‑IP in BV2 microglial cells. The Co‑IP results confirmed a direct physical interaction between TIPE2 and RhoA (Figure 5B). We then assessed the effects of TIPE2 on RhoA activation and downstream signalling in microglial cells. After stimulation with LPS and nigericin, we observed an increase in active RhoA (GTP‐RhoA) and ROCK2, a downstream effector, together with upregulated Glut1 and HK2, which linked to glycolysis. TIPE2 knockdown further enhanced these changes, whereas TIPE2 overexpression suppressed them (Figure 5C). To verify the status of RhoA activation, we detected membrane‑bound GTP‑RhoA using an active‑specific antibody. As shown in Figure 5D, TIPE2 knockdown promoted GTP‐RhoA movement toward the plasma membrane after LPS/nigericin challenge, while TIPE2 overexpression inhibited both its activation and membrane recruitment, consistent with the western blot results. Furthermore, we measured glucose uptake and lactate production in microglial cells. TIPE2 knockout significantly increased both parameters after LPS/nigericin stimulation, and this increase was effectively reversed by TIPE2 overexpression (Figure 5E and F). Quantitative statistical analysis plots of western blot data in Figure S2.

**FIGURE 5:**
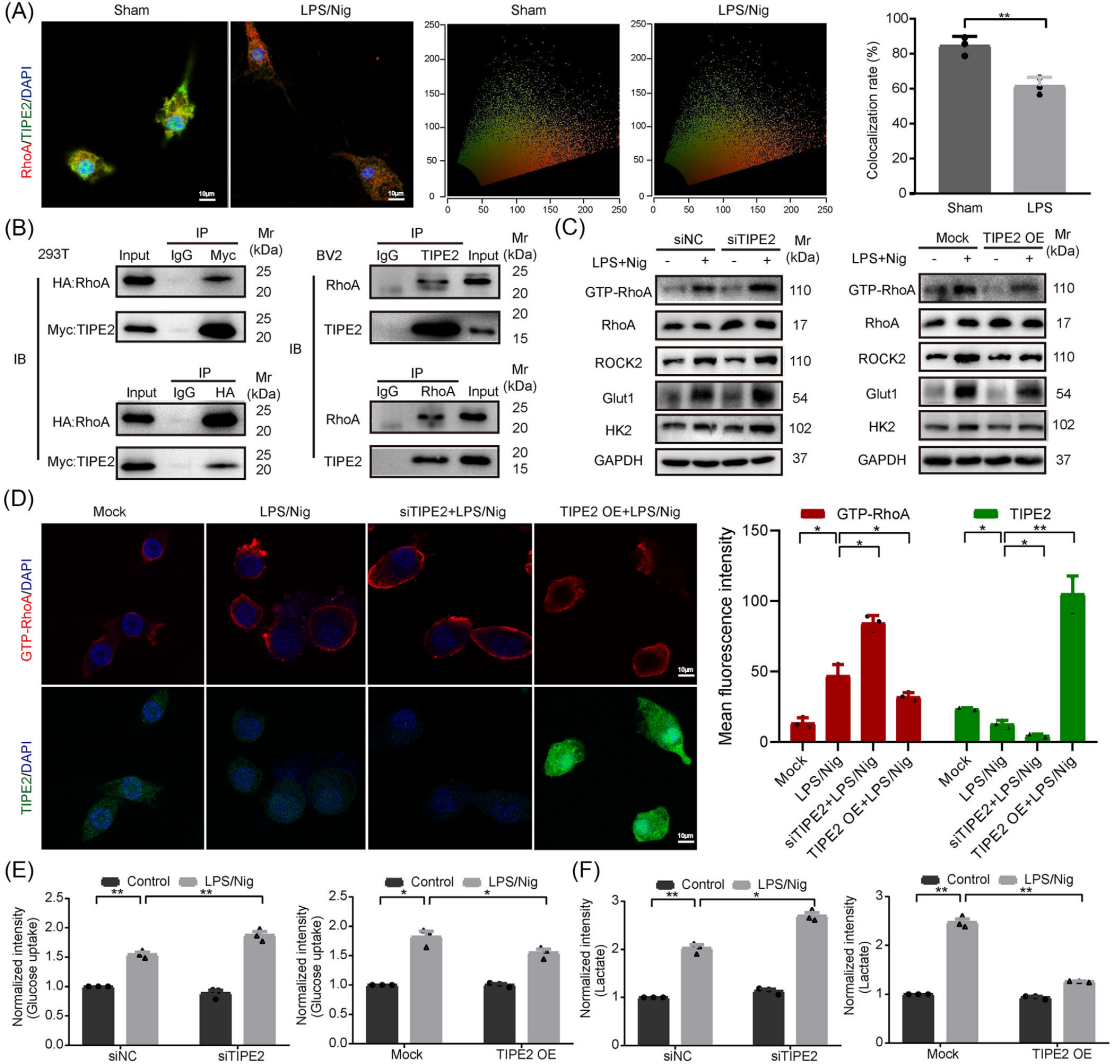
TIPE2 interacts with RhoA to inhibit RhoA/Glut1‐driven glycolysis. (A) Immunofluorescence shows co‐localisation of TIPE2 and RhoA. (B) The interaction of TIPE2 and RhoA was detected using Co‐IP assay in 293T cells and BV2 microglia. (C) The expression of ROCK2, GTP‐RhoA, Total‐RhoA, HK2 and Glut1 in BV2 microglia were determined after transfection of siTIPE2 and pCMV‐Myc‐TIPE2 plasmids by western blot. (D) Mean fluorescence intensity of TIPE2 (green) and GTP‐RhoA (red). Nuclei are stained with Hoechst (blue). Scale bar, 10 µm. (E) The glucose uptake of microglia was examined. (F) The amount of lactate produced by microglia was examined. *n* = 3 per group, *p < .05, **p < .01.

### 3.6 Rhosin reverses TIPE2 deficiency‑induced glycolysis and pyroptosis via RhoA inhibition

Primary microglia were further isolated for Western blot and ELISA analyses. The data demonstrated that in comparison with cell of WT+LPS/Nig group, TIPE2 KO+LPS/Nig samples exhibited enhanced RhoA activation, elevated expression of Glut1 and HK2, higher pyroptosis‐associated protein levels (NLRP3, Caspase‐1 p20, GSDMD‐N), and greater secretion of inflammatory cytokines. In contrast, treatment with the RhoA inhibitor Rhosin (TIPE2 KO+Rhosin+LPS/Nig) suppressed RhoA activation, reduced Glut1 and HK2 expression, and significantly attenuated both pyroptosis‑related protein levels and cytokine secretion (Figure 6A and B). Immunofluorescent staining showed that Rhosin suppressed ASC polymerisation (Figure 4C and E) and counteracted the increased iNOS (M1 marker) along with reduced Arg1 (M2 marker) caused by TIPE2 loss (Figure 4D and F). These data agreed with the immunoblot results (Figure 4G). These data indicate that the RhoA/Glut1 signalling pathway is involved in inflammasome activation and pyroptosis induced by TIPE2 deficiency. Furthermore, LPS/nigericin stimulation significantly increased glucose consumption, lactate production, and ROS generation in microglia, and these metabolic changes were reversed by Rhosin treatment in TIPE2 deficiency‑microglia (Figure 6H and I). Quantitative statistical analysis plots of western blot data in Figure S2.

**FIGURE 6:**
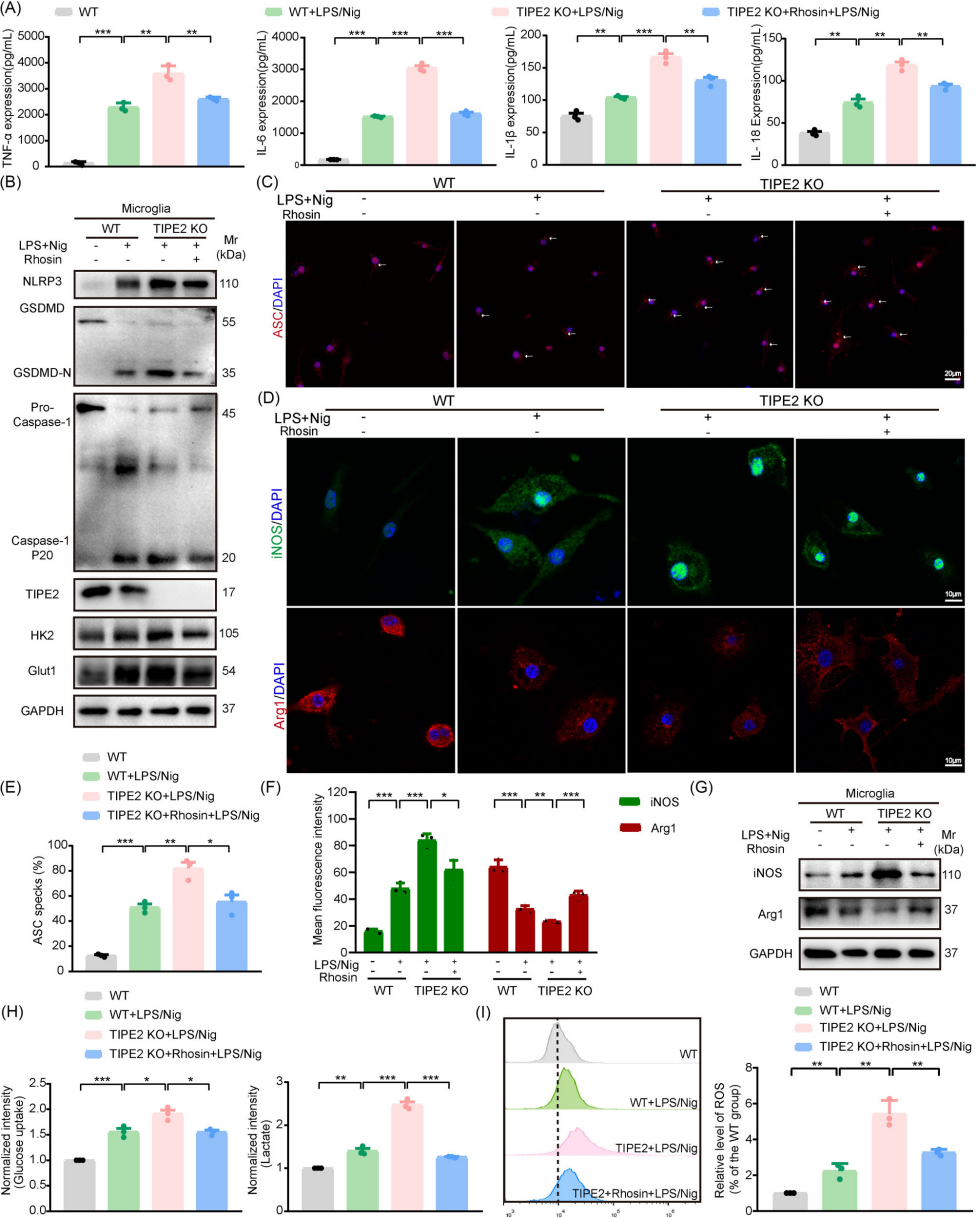
Rhosin reverses TIPE2 deficiency‑induced glycolysis and pyroptosis via RhoA inhibition. (A) Levels of TNF‐α, IL‐6, IL‐18 and IL‐1β in primary microglia supernatant were measured by ELISA. (B) The expression of TIPE2, NLRP3, Caspase‐1 P20, GSDMD‐N, HK2, Glut1 in primary microglia was determined by western blot. C and E) ASC was assessed by immunofluorescence. Scale bar, 20 µm. (D, F) The expression of microglial polarisation markers (iNOS and Arg1) in primary microglia were assessed by immunofluorescence. Scale bar, 10 µm. (G) The expression of iNOS and Arg1 in microglia were determined by western blot. (H) The glucose uptake and the amount of lactate produced by primary microglia was examined. (I) Levels of ROS in primary microglia. *n* = 3 per group, *p < .05, **p < .01, ***p < .001.

### 3.7 Rhosin alleviates TIPE2 deficiency‑exacerbated brain injury and cognitive deficits

Early pyroptotic cells exhibit apoptotic features such as chromatin condensation and TUNEL positivity; therefore, we performed TUNEL staining to evaluate neuronal apoptosis in vivo. As shown in Figure 7A, the TIPE2 KO+CLP group showed a higher apoptotic ratio than the WT+CLP group, and Rhosin treatment significantly ameliorated the TIPE2 deficiency‑exacerbated neuronal apoptosis in CLP‑induced mice. We further assessed synaptic ultrastructure and cognitive function using TEM and MWM. MWM data indicated that WT mice spent more time inside the target quadrant and had greater numbers of platform crossings after CLP. When comparing TIPE2 KO+Rhosin+CLP animals with TIPE2 KO+CLP animals, the former displayed reduced escape latencies across days 2–4, stayed considerably longer within the target quadrant, and crossed the platform more frequently(Figure 7B–E). Electron microscopy (Figure 7F) revealed that TIPE2 deficiency exacerbated synaptic injury in CLP mice, manifested by reduced postsynaptic membrane density and widened synaptic cleft, and this damaging trend was effectively counteracted by Rhosin. Immunoblot analysis revealed a marked reduction in PSD95 and SYP after CLP, which was worse in TIPE2‐deficient mice and Rhosin treatment improved these outcomes (Figure 7G and H). Collectively, these data indicate that Rhosin ameliorates cognitive impairment and neuronal injury aggravated by TIPE2 deficiency.

**FIGURE 7:**
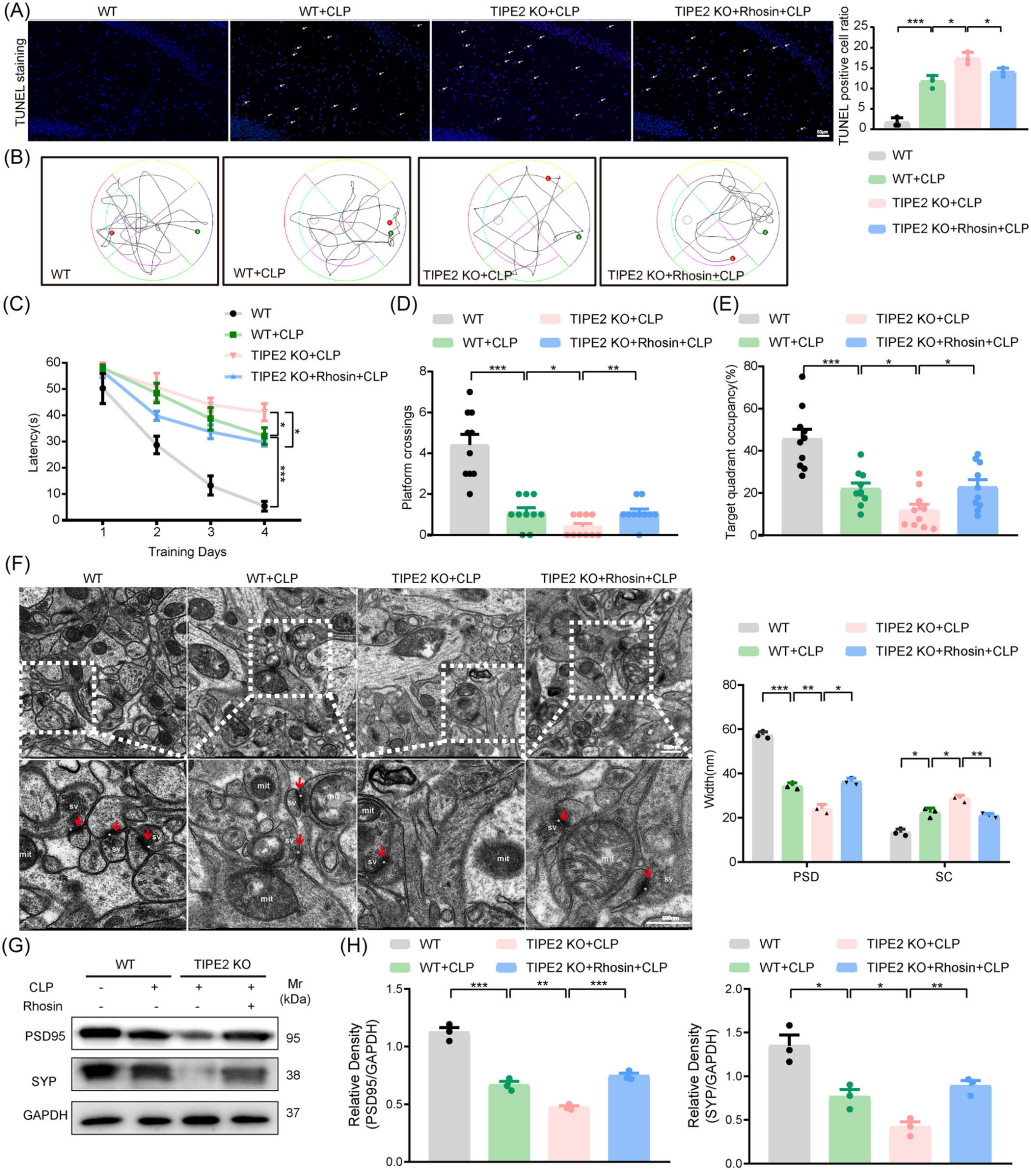
Rhosin alleviates TIPE2 deficiency‑exacerbated brain injury and cognitive deficits. (A) Proportion of apoptosis in the hippocampus of each group in TUNEL staining (*n* = 3 per group). Scale bar, 50 µm. (B) Representative swim traces of each group in the space exploration experiment on the 5th day (*n* = 10 per group). (C) Latency. The latency of each group in MWM tests for four consecutive days (*n* = 10 per group). (D) Platform crossings. Times of crossing the platform on the 5th day (*n* = 10 per group). (E) Target quadrant occupancy. The proportion of time spent in the target quadrant in the total time on the 5th day (*n* = 10 per group). (F) Ultrastructural changes in hippocampal synapses of each group under transmission electron microscopy (*n* = 3 per group). Scale bar, 500 nm. (G, H) Levels of PSD95 and SYP protein were measured by western blot (*n* = 3 per group). *p < .05, **p < .01, ***p < .001.

## 4 DISCUSSION

Neuroinflammation in the central nervous system (CNS) is a critical mechanism underlying sepsis‑induced brain dysfunction and delayed cognitive deficits, severely impairing the quality of life of SAE patients and increasing long‑term mortality.^15^ To date, the pathophysiological mechanisms of SAE remain poorly understood, and there is a lack of targeted clinical interventions. The activation of neuroinflammation in SAE predominantly occurs in microglia, where the amplified inflammatory cascade serves as a priming factor for cerebral damage.^16^ Therefore, targeted modulation of the inflammatory cascade within microglia holds significant potential for the early prevention and treatment of SAE. TIPE2, a novel negative regulator of immune homeostasis, plays an important role in various autoimmune and inflammatory diseases. It has been reported that TIPE2 reverses cognitive dysfunction induced by excessive inflammation in a mouse model of Alzheimer's disease.^17^, ^18^ Our previous research suggested that TIPE2 may inhibit the pathological progression of SAE neuroinflammation, although its precise molecular mechanism required further investigation. In this study, by combining single‑cell sequencing with a CLP‑induced SAE mouse model, we found that TIPE2 is specifically expressed in brain microglia and is significantly downregulated under SAE conditions. The hippocampus is responsible for much of the learning and memory processes and is also a region susceptible to neuroinflammation.^19^ Notably, TIPE2 knockout further exacerbated synaptic ultrastructural damage in the hippocampal region and cognitive dysfunction in SAE mice. However, the exact molecular mechanisms still need deeper exploration.

Microglia, as resident macrophages of the nervous system, play a key role in brain inflammation. During neuroinflammation, they release inflammatory mediators, leading to synaptic dysfunction and neuronal death.^20^ Studies have shown that M1‑polarised microglia predominate in the brains of septic patients.^21^ Inhibiting metabolic reprogramming can attenuate the surgical trauma‑induced shift of microglia toward the M1 phenotype, thereby improving neuroinflammation and cognitive dysfunction.^22^ In our in vivo study, we observed significant microglial activation in SAE mice, which was more pronounced in TIPE2‑knockout mice. By isolating primary microglia, we further found that TIPE2 knockdown promoted LPS‑induced microglial polarisation toward the M1 phenotype. To further investigate the role of TIPE2 in the dynamic functional changes of microglia in the SAE model and its underlying mechanisms, we performed single‐cell sequencing analysis on hippocampal cells collected from the CLP and sham groups. First, we performed clustering analysis based on cell identification features; we then found that TIPE2 was mainly expressed in microglia. Therefore, we further selected the microglial subpopulation for clustering analysis. Based on gene expression profiling, we found that one microglial cluster expressed higher levels of inflammation‐related genes than the other two clusters, and this cluster was significantly increased in the CLP group, suggesting that this subpopulation is closely associated with SAE. We named this cluster SAE‐associated microglia (SAM). Our previous study also identified SAM as a key microglial subpopulation involved in the development and progression of SAE.^23^ We then performed single‐cell RNA sequencing (scRNA‐seq) analysis on hippocampal cells isolated from WT and TIPE2 KO mice after CLP‐induced sepsis. The results indicated that TIPE2 deficiency may promote the generation or maintenance of pro‐inflammatory microglia, a process closely associated with pyroptosis and glycolysis. We performed single‑cell RNA‑sequencing (scRNA‑seq) analysis on hippocampal cells isolated from WT and TIPE2 KO mice following CLP‑induced sepsis. The analysis indicated that TIPE2 deficiency may promote the generation or maintenance of pro‑inflammatory microglia, which are closely associated with pyroptosis and glycolysis. Pyroptosis is a trigger for uncontrolled inflammation in sepsis. Sepsis activates microglia through peripheral inflammatory signals, leading to NLRP3 inflammasome activation and pyroptosis, thereby driving dysregulated inflammation.^24^, ^25^ Microglia are the primary sites of NLRP3‑mediated inflammasome hyperactivation and pyroptosis. Once the NLRP3 inflammasome is activated, downstream active proteins increase, resulting in pyroptosis and secretion of IL‑1β and IL‑18.^26^ Studies have demonstrated that LPS‑activated inflammasomes can accelerate neuroinflammation and induce synaptic defects.^27^, ^28^ By establishing knockout and overexpression models in primary microglia and BV2 cells, we demonstrated that TIPE2 deletion promotes IL‑1β release and pyroptosis, consequently exacerbating neuroinflammation. Mechanistically, TIPE2 deficiency activates the NLRP3 inflammasome and facilitates microglial pyroptosis, whereas TIPE2 overexpression suppresses these effects. Thus, TIPE2 deletion likely aggravates neuroinflammation by enhancing pyroptosis and cytokine release.

Studies have shown that glycolysis is essential for NLRP3 inflammasome activation in macrophages.^29^, ^30^, ^31^ This process enhances immune cell activation, microglial activation, and pro‑inflammatory cytokine release, contributing to brain inflammation and neuronal damage.^32^, ^33^ In line with these reports, we found that TIPE2 deficiency promotes glycolysis in LPS‑stimulated microglia, as evidenced by upregulation of glycolysis‑related proteins, increased glucose consumption, and lactate production, which was also supported by our scRNA‑seq data. In recent years, the involvement of Rho proteins in the regulation of infection and inflammation has gained increasing attention.^34^ RhoA activation is particularly important in CNS injury mechanisms; high levels of RhoA expression and activity have been observed in traumatic brain injury and spinal cord injury.^35^, ^36^ Persistent RhoA activity and expression after CNS injury may limit CNS regeneration and functional recovery.^37^ Hence, targeting RhoA‑mediated signal transduction may be an effective strategy to mitigate sepsis‑induced neurological damage. Importantly, RhoA has been shown to participate in insulin‑stimulated Glut4 translocation and to regulate glucose uptake; activated RhoA promotes Glut1 translocation to the plasma membrane to stimulate glycolysis.^38^, ^39^ This further suggests that RhoA‑regulated signalling may serve as a potential target for microglial glycolytic reprogramming under inflammatory conditions. In this study, CO‐IP experiments revealed that TIPE2 can directly bind to RhoA, thereby inhibiting downstream NLRP3 expression and negatively regulating the pyroptosis pathway in microglia. However, the specific binding sites of TIPE2 and rhoA still require further investigation. When we analysed the transcriptional gene expression differences in the SAM subset before and after TIPE2 knockout, we did not find a significant difference in RhoA expression in the SAM subset after TIPE2 knockout, whereas the expression of its downstream effector gene ROCK2 was significantly upregulated. We believe that this phenomenon may be because RhoA activity is mainly regulated at the post‐translational level through GTP loading. Upon stimulation, RhoA translocated from the cytoplasm to the cell membrane and binds GTP to convert into the active form GTP‐RhoA; GTP‐RhoA then interacts with downstream effector proteins to exert biological effects. However, changes in GTP‐RhoA cannot be captured by scRNA‐seq. Nevertheless, by using glutathione affinity beads conjugated with GST‐fused Rhotekin‐RBD to capture GTP‐RhoA bound to Rhotekin‐RBD, and by using immunofluorescence to directly detect GTP‐RhoA expression on the cell membrane, we can both found that TIPE2 knockout inhibits RhoA activation. In addition, our experimental results demonstrate that TIPE2 regulates microglial pyroptosis and glycolytic reprogramming. Following LPS/Nigericin treatment, the RhoA/Glut1 pathway was overactivated in TIPE2‐knockdown microglia with upregulated Glut1 and HK2 expression, increased glucose uptake and lactate production, pyroptosis of microglia subsequently increased whereas TIPE2 overexpression produced the opposite effects. Notably, pharmacological inhibition of RhoA activation with a specific inhibitor (Rhosin) significantly reversed both enhanced microglial pyroptosis and glycolysis induced by TIPE2 deficiency. Together with our co‐immunoprecipitation and immunofluorescence data, these findings collectively support the conclusion that TIPE2 regulates microglial pyroptosis and glycolysis through the RhoA/Glut1 pathway. However, the specific molecular mechanisms by which RhoA mediates these effects has yet to be completely clarified. This question also represents a key focus for upcoming investigations in our laboratory. Furthermore, we observed that TIPE2 deficiency markedly increased ROS production in LPS/nigericin‑stimulated microglia, whereas TIPE2 overexpression suppressed ROS elevation. Previous studies have shown that ROS are not merely markers of oxidative stress but also critical signalling molecules linking metabolic reprogramming to inflammasome activation. ROS promote NLRP3 inflammasome activation through multiple mechanisms: on one hand, ROS directly mediate the dissociation and binding of thioredoxin‑interacting protein (TXNIP) with NLRP3;^40^, ^41^ on the other hand, ROS‑induced mitochondrial dysfunction leads to the release of mitochondrial DNA, further activating NLRP3.^42^, ^43^ Moreover, enhanced glycolysis itself can promote the activation of NADPH oxidases (e.g., NOX2), generate more ROS and establish a positive feedback loop of ‘glycolysis–ROS–inflammasome’.^44^, ^45^ Thus, the TIPE2/RhoA/Glut1 axis may indirectly reduce ROS bursts by inhibiting glycolytic flux, thereby blocking persistent NLRP3 inflammasome activation.

Based on these findings, we propose that TIPE2 acts as an upstream regulator of RhoA. By interacting with and inhibiting RhoA activation, TIPE2 subsequently blocks the RhoA/Glut1 signalling pathway. This mechanism ultimately alleviates microglial pyroptosis and glycolysis, attenuates hippocampal neuronal damage, and improves cognitive dysfunction in SAE. Nevertheless, there are still some limitations in the content and details of this part of the study. First, our study has examined solely the contribution of microglia to SAE‐associated neuroinflammation, whereas exploring the interactions among different neural cell types during SAE pathology also holds significant research value. This represents a potential direction for our future studies, in which we can further investigate the crosstalk or dialogue between microglia and astrocytes in regulating their respective processes. In addition, in the animal part of this study, we only demonstrated that TIPE2 knockout aggravates neurological injury and cognitive dysfunction in SAE mice; more definitive therapeutic effects still require auxiliary evidence from TIPE2 overexpression. Although we have already performed relevant validation studies on TIPE2 overexpression in cell experiments, overexpressing TIPE2 in the hippocampus via AAV delivery for overexpression validation would further strengthen our conclusions—this will be a direction for future validation.

## AUTHOR CONTRIBUTIONS

ZYX, XJW, and XMS conceived and designed the study, reviewed the data, and revised the manuscript. MY, YZY, and GQJ performed the experiments. MY, QK, and YXW processed the experimental data and prepared the manuscript figures. HFL and HLG wrote the main part of the manuscript. WHL, ZYZ, and MY discussed the results and reviewed the manuscript. All authors read and approved the final manuscript.

## CONFLICT OF INTEREST STATEMENT

The authors declare no conflicts of interest.

## CONSENT FOR PUBLICATION

All authors have read and approved the final version of the manuscript and agree with its submission to *Clinical and Translational Medicine*.

## ETHICS STATEMENT

All animal care and experiments were performed according to the Institutional Animal Care and Use Committee of Wuhan University. The study protocol was approved by the Animal Experimentation Committee of Wuhan University (WQ20220005).

## Supporting information



Supporting Information

## Data Availability

Data and materials are available upon reasonable request if applicable.
