# Supplementary material for: Regulatory role of TIPE2 by inhibiting microglial pyroptosis and glycolysis in the treatment of sepsis‐associated encephalopathy
Source: Clin Transl Med. 2026 Nov 6;16(11):e70881. doi: 10.1002/ctm2.70881 (PMC13741204; doi:10.1002/ctm2.70881)
Supplement: Supplementary file 1 [file CTM2-16-e70881-s001.docx]

**Supplementary Information**

**Figure S1.** (**A**)Generation and identification of TIPE2 KO mice. (**B**) The expression of TIPE2 in TIPE2 KO mice and WT mice by western blotting. (**C**) The expression of TIPE2 in BV2 cell with siNC or siTIPE2 in different concentrations (5,30,50nM) by western blotting. (**D**) The expression of TIPE2 in BV2 cell with siTIPE2 or pCMV-Myc-TIPE2 plasmid by western blotting. (**E**) The expression of GTP-RhoA in BV2 cell with rhosin in different concentrations (0,5,10,20,30μM) by western blotting.

**Figure S2.** Quantitative statistical analysis plots of western blot data in Figure 4, Figure 5 and Figure 6.

**Figure S2.** Immunofluorescence staining of TIPE2 (green) and GTP-RhoA (red) in Figure 6D with separation and merge channels. Nuclei are stained with Hoechst (blue). Scale bar, 10 μm.
